# Carbon/Graphene-Modified Titania with Enhanced Photocatalytic Activity under UV and Vis Irradiation

**DOI:** 10.3390/ma12244158

**Published:** 2019-12-11

**Authors:** Kunlei Wang, Maya Endo-Kimura, Raphaëlle Belchi, Dong Zhang, Aurelie Habert, Johann Bouclé, Bunsho Ohtani, Ewa Kowalska, Nathalie Herlin-Boime

**Affiliations:** 1 Institute for Catalysis (ICAT), Hokkaido University, N21 W10, Sapporo 001-0021, Japan; kunlei@cat.hokudai.ac.jp (K.W.); m_endo@cat.hokudai.ac.jp (M.E.-K.); ohtani@cat.hokudai.ac.jp (B.O.); 2 IRAMIS—NIMBE UMR 3685, Université Paris Saclay, CEA Saclay, 91191 Gif/Yvette CEDEX, France; raphaelle.belchi@cea.fr (R.B.); aurelie.habert@cea.fr (A.H.); 3 Univ. Limoges, CNRS, XLIM, UMR 7252, F-87000 Limoges, France; johann.boucle@unilim.fr; 4 Graduate School of Environmental Science, Hokkaido University, Sapporo 060-0810, Japan; zhang.d@cat.hokudai.ac.jp

**Keywords:** carbon-doped titania, carbon-modified titania, graphene/titania, vis-active photocatalyst, antibacterial properties, laser pyrolysis

## Abstract

Laser synthesis was used for one-step synthesis of titania/graphene composites (G-TiO_2_ (C)) from a suspension of 0.04 wt% commercial reduced graphene oxide (rGO) dispersed in liquid titanium tetraisopropoxide (TTIP). Reference titania sample (TiO_2_(C)) was prepared by the same method without graphene addition. Both samples and commercial titania P25 were characterized by various methods and tested under UV/vis irradiation for oxidative decomposition of acetic acid and dehydrogenation of methanol (with and without Pt co-catalyst addition), and under vis irradiation for phenol degradation and inactivation of *Escherichia coli*. It was found that both samples (TiO_2_(C) and G-TiO_2_(C)) contained carbon resulting from TTIP and C_2_H_4_ (used as a synthesis sensitizer), which activated titania towards vis activity. The photocatalytic activity under UV/vis irradiation was like that by P25. The highest activity of TiO_2_(C) sample for acetic acid oxidation was probably caused by its surface enrichment with hydroxyl groups. G-TiO_2_(C) was the most active for methanol dehydrogenation in the absence of platinum (ca. five times higher activity than that by TiO_2_(C) and P25), suggesting that graphene works as a co-catalyst for hydrogen evolution. High activity under both UV and vis irradiation for decomposition of organic compounds, hydrogen evolution and inactivation of bacteria suggests that laser synthesis allows preparation of cheap (carbon-modified) and efficient photocatalysts for broad environmental applications.

## 1. Introduction

Titania (titanium(IV) oxide; titanium dioxide) is probably the most intensively studied semiconductor for both environmental and energy application, i.e., water/air purification, wastewater treatment, self-cleaning surfaces, sterilization, photocurrent generation, water splitting and fuel production [1,2,3,4,5,6]. Although titania has many advantages, including low-cost, high activity and stability, two main shortcomings limit titania broad and common applications. First, quantum yields of photocatalytic reactions driven by titania are much lower than 100% since photogenerated charge carriers recombine fast, as typical for all semiconductors. Moreover, wide bandgap (ca. 3 eV, depending on polymorphic forms) of titania allowing good redox properties results in its activity only under UV irradiation, and thus main part of solar radiation cannot be used for photocatalytic process.

Therefore, various methods have been proposed for titania modifications, such as doping (substitutional or/and interstitial), surface modification or formation of composites with other materials (e.g., heterojunctions). The most famous and probably most efficient for activity enhancement under UV irradiation is surface modification of titania with noble metals, since noble metals work as an electron sink (larger work function than electron affinity of titania), and thus hindering charge carriers’ recombination, as probably firstly reported by Kraeutler and Bard more than forty years ago [7]. Recently, another property of noble metals, i.e., surface plasmon resonance (SPR) at vis range has been studied to activate titania towards vis irradiation [8,9]. However, noble metals are quite expensive, and thus cheaper materials are more recommended. For example, titania modification with carbon seems to be more attractive for broad and commercial application.

Various sources of carbon have been proposed for titania modification, such as polyvinyl alcohol [10], n-hexane [11], alcohols (methyl, ethyl, isopropyl, n-butyl, 2-butyl and tert-butyl alcohols) [12], benzene [13], ethylene glycol and pentaerythritol [14] and glucose [15]. Interesting approach was proposed for carbon-doped titania nanostructures (micro- and nanospheres and nanotubes), synthesized via a single source vapor deposition in an inert atmosphere (Ar), where titanium butoxide (common organic precursor of titania) was used also as a carbon source [16]. Similar findings were reported for titania prepared from titanium isopropoxide by the sol–gel method and calcined at different temperatures (350, 450, 550, 650 and 750 °C) [17]. It was found that calcination at 350 °C resulted in the strongest vis absorption, correlating with the highest surface content of carbon and the highest photocatalytic activity under vis irradiation. However, surface modification (with C–C species) was suggested as the main reason of vis response, rather than C-doping (no bandgap narrowing).

Recently, 2D carbon structures, i.e., graphene (G), graphene oxide (GO) and reduced graphene oxide (rGO), have been proposed for titania modification, due to large specific surface area (efficient reagents adsorption), high conductivity (inhibited charge carriers’ recombination by highly mobile electrons), flexible structure and high stability [18]. For example, (i) TiO_2_-GO composite, prepared by thermal hydrolysis of suspension containing GO and titania peroxo-complex, was efficient for photocatalytic degradation of butene in the gas phase [19], (ii) TiO_2_-rGO, synthesized by ionothermal method, was able to generate hydrogen [20], (iii) hydrothermally prepared TiO_2_-rGO and TiO_2_-G decomposed bisphenol A under both UV and vis irradiation [21] and 4-chlorophenol under solar radiation [22], respectively, (iv) ZnO-G, prepared by the Hummers/Offeman/hydrothermal method, caused efficient degradation of cyanide in water under UV, vis, solar and even NIR irradiation (plasmonic absorption of NIR through coupling of graphene) [23] and (v) α-Fe_2_O_3_-ZnO/rGO, synthesized by the Hummer method, redox replacement and electrochemical process, was able to capture and reduce CO_2_ to CH_3_OH under vis irradiation [24]. Accordingly, G, GO and rGO-modified titania samples are widely used for photocatalytic applications. However, the role of 2D carbon has not been completely clarified yet, as summarized in recent review paper by Giovannetti et al. [18]. It was proposed that under UV irradiation, electrons from conduction band (CB) of titania migrate to graphene, due to its more positive Fermi level, which hinders charge carriers’ recombination. Whereas, under vis irradiation, the opposite direction of electrons’ transfer has been proposed, i.e., from photoexcited state of graphene to CB of titania.

Our recent study on graphene-modified titania has shown that this material might be efficiently used for perovskite solar cells, where graphene presence enhanced power conversion efficiency significantly, as a result of photoluminescence quenching, due to enhanced electron migration [25]. Therefore, to further examine the potential and application possibility of graphene-modified titania, prepared by a novel and simple method using laser pyrolysis, more detailed characterization and photocatalytic activity for degradation of organic compounds, hydrogen evolution and microorganism inactivation under both UV and vis irradiation have been investigated in this study.

## 2. Materials and Methods

### 2.1. Synthesis

**Figure 1:**
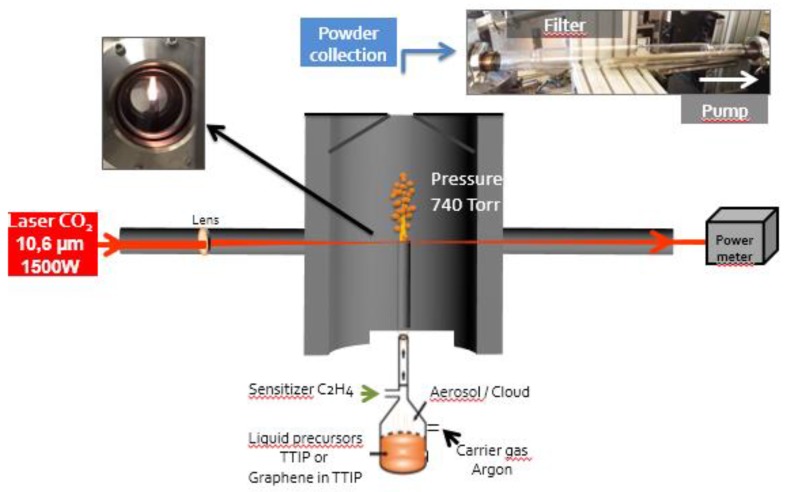
Schematic drawing of the laser pyrolysis experimental set-up for preparation of titania samples (TiO_2_(C) from TTIP and G-TiO_2_(C) from TTIP and graphene).

Titania (TiO_2_(C)) and graphene-modified titania (G-TiO_2_(C)) were prepared by laser pyrolysis. This synthesis technique has already been described in several publications, e.g., Pignon et al. for the synthesis of TiO_2_ with controlled anatase to rutile ratio and Belchi et al. for the one-step synthesis of graphene/TiO_2_ nanocomposites [25,26]. A schematic drawing of the experimental set-up is shown in Figure 1, and the most salient features of the technique are described hereafter. Briefly, liquid titanium(IV) tetraisopropoxide (TTIP) purchased from Sigma-Aldrich (≥97% purity) was used for the synthesis of titania (labeled as TiO_2_(C)). A suspension of 0.04 wt% industrial graphene (reduced graphene oxide G-200 from SIMBATT Company (Shanghai, China), oxygen content of less than 8 at%, a few layers (<10 layers), leading to a high specific area (>600 m^2^/g)) dispersed in TTIP was used for the synthesis of graphene-TiO_2_(C) nanocomposite material labeled as G-TiO_2_(C). Droplets of the liquid precursor or suspension were obtained with a pyrosol device (RBI, Meylan, France). The droplets were carried out with a carrier gas (Ar) to the reactor where they intersected with the beam of a high-power CO_2_ laser (maximum emission at 10.6 μm). TTIP could not absorb well the laser radiation, therefore C_2_H_4_ was added as a sensitizer to the Ar carrier gas. After laser absorption, the reactive medium was rapidly thermalized by collisional transfer. Decomposition of precursors and growth of nanoparticles occurred with an appearance of a flame.

The laser internal power was set at 530 W corresponding to a 400 W laser power, measured under Ar atmosphere after the reaction zone. The laser power measured at the same place during the reaction was 230 W. The pressure was regulated at atmospheric pressure, i.e., 10^5^ Pa. The ethylene (sensitizer) and argon (carrier) gas flow rates were 355 and 2000 sccm, respectively. The production rate was 1.3 g/h for the TiO_2_(C) and 0.36 g/h for the G-TiO_2_(C) sample. This difference is caused by the higher viscosity of the suspension in the presence of graphene, leading to reduced generation of droplets by the pyrosol device. Post-reaction annealing (6-h under air at 450 °C) was performed to remove amorphous carbon present in the powders, due to the carbon content in the precursors.

### 2.2. Characterization of Samples

The morphology of the powders was evaluated by a Carl Zeiss ULTRA55 scanning electron microscope (Carl Zeiss, Oberkochen, Germany) and by a JEOL 2010 high-resolution transmission electron microscope (Jeol, Tokyo, Japan) operated at 200 kV. For SEM analysis, the powder was directly observed on the carbon tape. For HRTEM measurements, the powder was dispersed in ethanol and nanoparticles were separated with intensive ultrasound radiation using a Hielscher Ultrasound Technology VialTweeter UIS250V (Teltow, Germany). Then, the dispersion was dropped on a grid made of a Lacey Carbon Film (300 mesh Copper, S166-3H, purchased from Oxford instruments SAS, Abingdon, Great Britain). The Raman spectra were acquired on an XploRA PLUS Horiba apparatus (Kyoto, Japan) using a laser emission at 532 nm for excitation. The composition of samples was estimated by an energy-dispersive X-ray spectroscopy (EDS; HD-2000, HITACHI, Tokyo, Japan).

Photoabsorption properties for samples before and after annealing were analyzed by diffuse reflectance spectroscopy (DRS; JASCO V-670 equipped with a PIN-757 integrating sphere, JASCO, LTD., Pfungstadt, Germany). Barium sulfate was used as reference for DRS analysis. Crystalline properties were analyzed by X-ray powder diffraction (XRD; Rigaku intelligent XRD SmartLab with a Cu target, Rigaku, LTD., Tokyo, Japan). Crystallite sizes of anatase, rutile and graphene were estimated using the Scherrer equation. Chemical composition of the surface (content and chemical state of elements, i.e., titanium, oxygen and carbon) was determined by X-ray photoelectron spectroscopy (XPS; JEOL JPC-9010MC with MgKα X-ray, JEOL, LTD., Tokyo, Japan).

Energy-resolved distribution of electron traps (ERDT) pattern and conduction band bottom (CBB) position were analyzed by reversed double-beam photoacoustic spectroscopy (RDB-PAS) and photoacoustic spectroscopy (PAS), respectively, and detailed procedure was described elsewhere [27]. In brief, for RDB-PAS measurement, as-prepared sample was filled in stainless-steel sample holder in a home-made PAS cell, equipped with an electret condenser microphone and a Pyrex window on the upper side. Methanol-saturated nitrogen was flowed through the cell for 30 min, the cell was irradiated by a 625-nm light-emitting diode beam (Luxeon LXHL-ND98, LUMILEDS, San Jose, CA, USA), modulated at 35 Hz by a function generator (DF1906, NF Corporation, Yokohama, Japan) as modulated light, and a monochromatic light beam from a Xe lamp (ASB-XE-175, Spectral Products, Putnam, CT, USA), equipped with a grating monochromator (CM110 1/8 m, Spectral Products, CT, USA) as continuous light. The continuous light was scanned from 650 to 300 nm with a 5-nm step. RDB-PA signal was detected by a digital lock-in amplifier (LI5630, NF Corporation). Obtained spectrum was differentiated from the lower-energy side and calibrated with the reported total electron-trap density in units of μmol g^−1^ measured by a photochemical method [28] to obtain an ERDT pattern. For PAS measurements, the cell window was irradiated from 650 to 300 nm by a light beam from a Xe lamp (ASB-XE-175, Spectral Products) with a grating monochromator (CM110 1/8m, Spectral Products, CT, USA) modulated at 80 Hz by a light chopper (5584A, NF Corporation, Japan) to detect the PAS signal using a digital lock-in amplifier, and then photoacoustic (PA) spectra were calibrated with a reference of a PA spectrum of graphite. The CBB, as energy from the top of the valence band (VBT), of samples was calculated from the onset wavelength corresponding to the bandgap of samples.

### 2.3. Activity Tests

The photocatalytic activity of samples was evaluated under UV/vis irradiation for: (1) oxidative decomposition of acetic acid (CO_2_ evolution) and (2) anaerobic dehydrogenation of methanol (H_2_ evolution; with/without in situ platinum deposition (2 wt% in respect to TiO_2_), photodeposition details might be found here [29]), and under vis irradiation for (3) oxidation of phenol. For activity tests, (1–2) 50 mg and (3) 10 mg of photocatalyst was suspended in 5 mL of aqueous solution of (1) acetic acid (5 vol%), (2) methanol (50 vol%) and (3) 0.21 mmol/L phenol in 35-mL Pyrex test tubes. The tubes were sealed with rubber septa, the suspensions were continuously stirred in a thermostated water bath and irradiated by (1–2) Hg lamp (λ > 290 nm) and Xe lamp (λ > 420 nm; Xe lamp, water IR filter, cold mirror and cut-off filter Y45, in a reactor shown in Figure 3 of Ref. [30]). For methanol dehydrogenation, two kinds of reactions were carried out, i.e., with (“H_2_ system (Pt)”) and without (“H_2_ system (no Pt)”) platinum deposited in situ and working as co-catalyst for hydrogen formation. Suspensions containing titania and methanol and hexachloroplatinic acid (H_2_PtCl_6_ 6H_2_O; in the case of “H_2_ system (Pt)”) were pre-bubbled with Ar (100 mL/min, 15 min) to remove oxygen from the system. The amounts of (1) generated hydrogen, (2) liberated carbon dioxide and (3) phenol and benzoquinone (main degradation product) were determined by chromatography: (1–2) gas chromatography with thermal conductivity detector (GC-TCD), gas phase sampled every 20 min for (1) or 15 min for (2)) and (3) HPLC (liquid phase sampled every 30 min).

Antibacterial activity was evaluated using *Escherichia coli* K12 (ATCC29425, Manassas, VA, USA) as a model of bacteria. The procedure was described elsewhere [31]. In brief, 10 mg of sample was suspended in 7 mL of bacterial suspension (ca. 1–5 × 10^8^ cells/mL) in Pyrex-glass test tube and irradiated with xenon lamp, equipped with cold mirror and cut-off filter Y45 (λ > 420 nm) or kept in the dark under continuous stirring at 25 °C. After irradiation at 0.5, 1, 2 and 3 h, portions of suspension were taken, diluted and inoculated on the plate count agar (Becton, Dickinson and Company, Franklin Lakes, NJ, USA) medium. Agar plates were incubated at 37 °C for ca. 16 h, and then formed colonies were counted.

Commercial titania P25 (Degussa/Evonik) was used as reference sample since P25 has exhibited one of the highest photocatalytic activity among various titania samples in different reaction systems (oxidation and reduction) [29,32,33], and thus is commonly used as a “standard” titania sample.

## 3. Results and Discussion

### 3.1. Characterization of Samples

**Figure 2:**
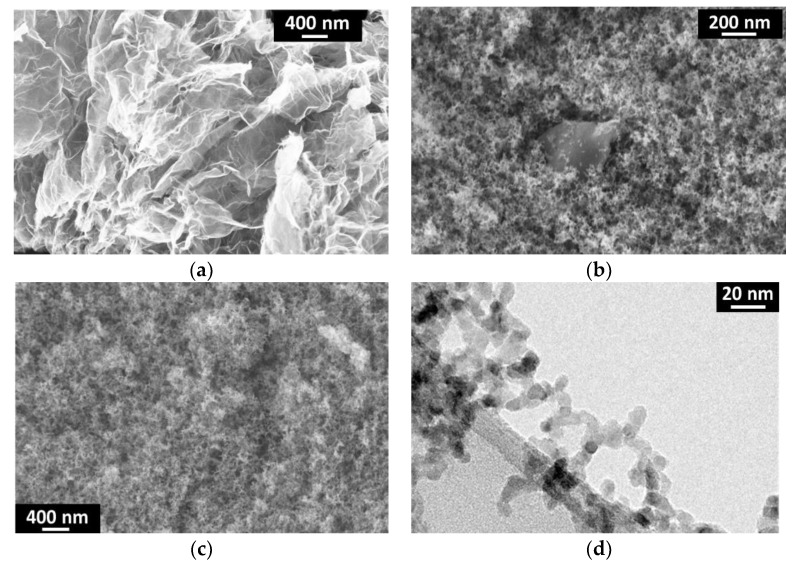
SEM (**a**–**c**) and TEM (**d**) images of: (**a**) graphene (SIMBATT); (**b**) G-TiO_2_(C) and (**c**,**d**) TiO_2_(C) samples.

SEM and TEM images of graphene, TiO_2_(C) and G-TiO_2_(C) samples are given in Figure 2 Original graphene sheets and graphene sheets covered with fine NPs of titania are shown in Figure 2a,b, respectively. Figure 2c,d illustrates TiO_2_(C) sample, with good homogeneity of fine TiO_2_ NPs of ca. 10–20 nm, corresponding well with the diameter estimated from specific surface area (SSA) measurement by BET (Brunauer, Emmet and Teller) method (ca. 25 nm).

**Figure 3:**
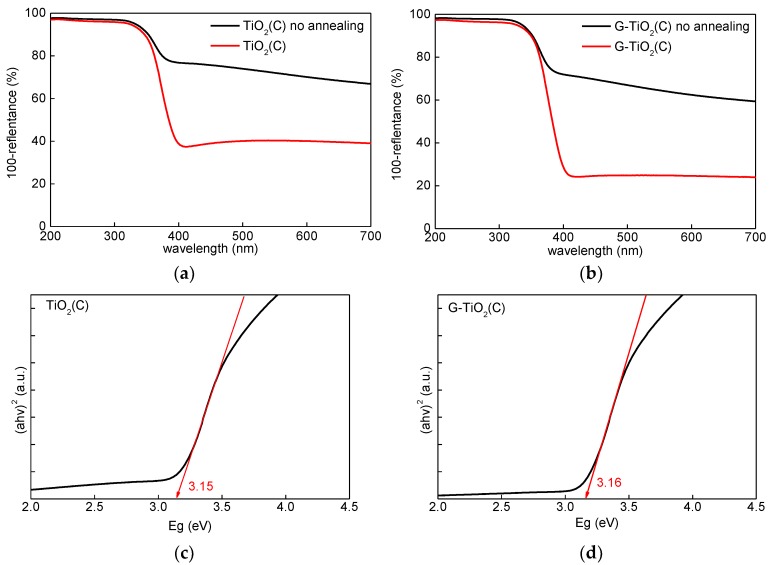
(**a**,**b**) Diffuse reflectance spectroscopy (DRS) spectra of samples before (black) and after (red) annealing: (**a**) TiO_2_(C) and (**b**) G-TiO_2_(C); (**c**,**d**) Bandgap estimation using Tauc’s bandgap plots for samples after annealing: (**c**) TiO_2_(C) and (**d**) G-TiO_2_(C).

The samples formed by the laser pyrolysis were grey, due to the presence of significant content of carbon (ca. 25% [25]) from titania precursor (TTIP) and C_2_H_4_ (a pyrolysis sensitizer). Although the previous study indicates the disappearance of the grey color after annealing (430 °C for 6 h; The conditions were estimated carefully to remove carbon impurities, and to avoid graphene combustion [25]), annealed samples were still greyish, probably due to different geometric conditions in a new furnace (KDF S-70; Denken-High Dental Co., Ltd.). The respective photoabsorption spectra before and after annealing are shown in Figure 3a,b.

Similar photoabsorption spectra have already been reported for C-modified titania, prepared from TTIP by the sol–gel method and calcination at 350 °C [17]. Interestingly, it was found that photoabsorption properties at vis range were stronger for TiO_2_(C) sample than for G-TiO_2_(C) sample (both before and after annealing), which suggests that annealing resulted in removal of only part of the carbon used for synthesis. It is possible that adsorption of titania NPs on graphene sheets resulted in less available area of titania surface to be modified with carbon (from TTIP and C_2_H_4_). It should be pointed that thermal treatment (annealing) might cause stable surface modification of titania with carbon [17], whereas the pyrolysis might result in carbon doping (since carbon is introduced during titania synthesis), similar to samples prepared via vapor deposition [16]. Therefore, both kinds of modification could be expected in the case of TiO_2_(C) sample, i.e., C-doping and C-modification. Whereas, in the case of G-TiO_2_(C), titania was additionally modified with graphene. Although, clear bandgap narrowing was not observed, it is proposed that C-doping might be possible, considering slight shift of absorption edge from 388 nm (for pure anatase) [34] towards vis region, as calculated from bandgap energy [35] (Figure 3c,d)), i.e., ca. 394 nm (E_g_ = 3.15 eV) and 392 nm (E_g_ = 3.16 eV) for TiO_2_(C) and G-TiO_2_(C) samples, respectively.

**Figure 4:**
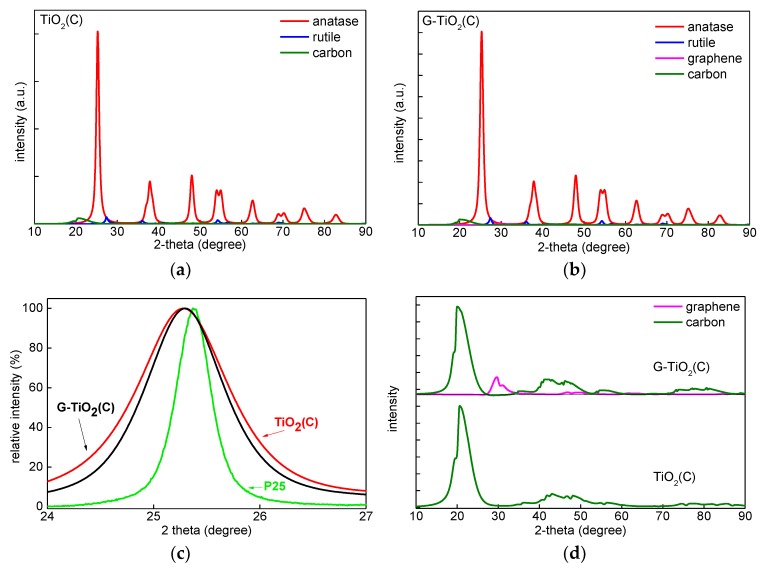
XRD patterns of: (**a**) TiO_2_(C); (**b**) G-TiO_2_(C), (**c**) TiO_2_(C), G-TiO_2_(C) and P25 titania at (101) anatase peak; (**d**) G-TiO_2_(C) and TiO_2_(C) after subtraction of titania peaks.

**Figure 5:**
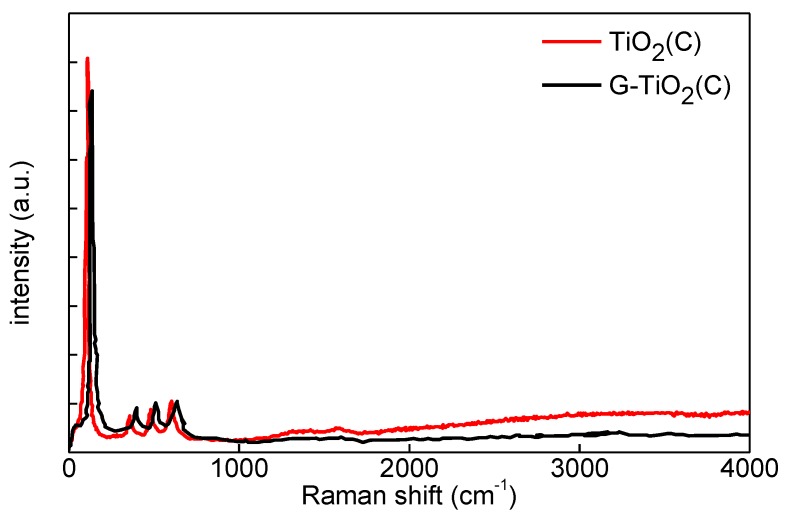
Raman spectra of TiO_2_(C) (red) and G-TiO_2_(C) (black).

**Figure 6:**
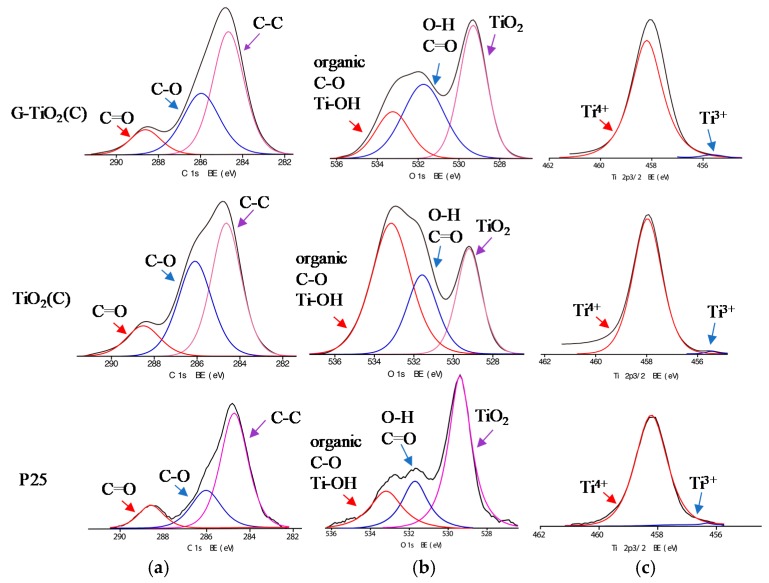
XPS data for G-TiO_2_(C) (top) and TiO_2_(C) (middle) and P25 (bottom) samples for: (**a**) C 1s, (**b**) O 1s and (**c**) Ti 2p_3/2_.

**Figure 7:**
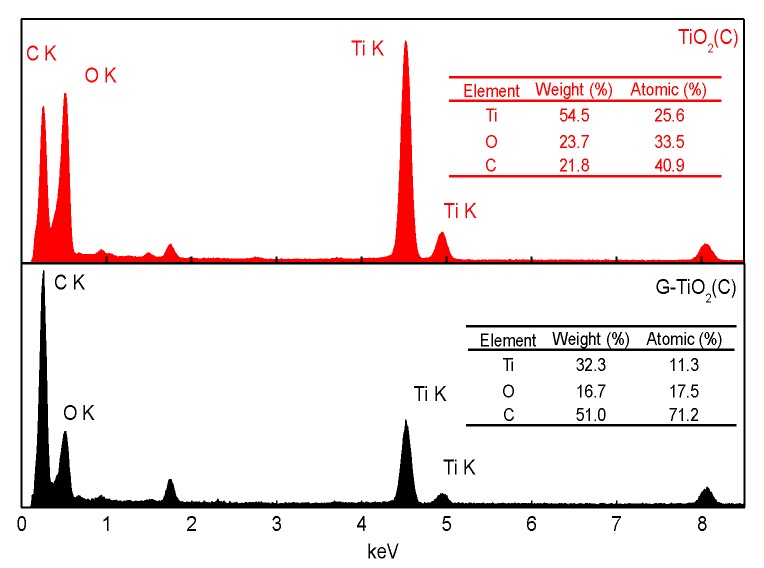
EDS data for TiO_2_(C) (red, upper) and for G-TiO_2_(C) (black, down).

**Figure 8:**
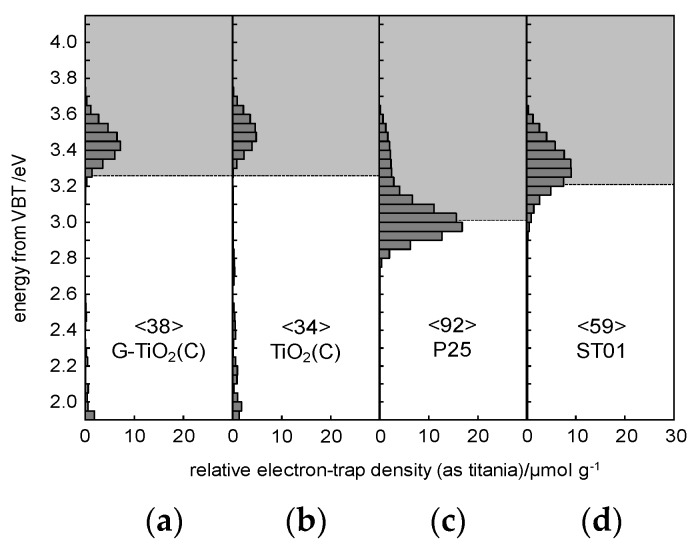
Energy-resolved distribution of electron traps (ERDT) pattern (bars) and conduction band bottom (CBB) position (bottom of grey box) of: (**a**) G-TiO_2_(C), (**b**) TiO_2_(C), (**c**) P25 and (**d**) ST01; values in < > denote the total density of electron traps (ETs) in the unit of μmol g^−1^.

To find the reason of vis absorption by TiO_2_(C) sample, and to characterize both samples in detail, XRD, XPS, EDS, Raman spectroscopy and PAS/RDB-PAS analyses were performed, and obtained data are shown in Figure 4, Figure 5, Figure 6, Figure 7 and Figure 8 XRD patterns of both samples were very similar, indicating that anatase was the main crystalline form, i.e., 78.7% in TiO_2_(C) and 79.2% in G-TiO_2_(C) (Figure 4 and Table 1). The minority of rutile was also detected in both samples, reaching 4.3% in TiO_2_(C) and 3.6% in G-TiO_2_(C), and thus the ratios of anatase to rutile were 18.5:1 and 22:1, respectively. The smaller content of rutile in G-TiO_2_(C) sample suggests that co-present graphene could inhibit the anatase-to-rutile phase transition during annealing. The graphene was detected only in G-TiO_2_(C) sample (ca. 3.9%). Although the signal was not intense, clear pattern could be seen after subtraction of titania peaks, as shown in Figure 4d (pink). It was found that the typical diffraction peak of graphene at 2*θ* = 26.7° [21,36] was shifted to ca. 30° (at max. intensity) indicating possible partial hybridization with titania. Moreover, the additional, broad peaks at ca. 22° (002), 40–50° ((100), (101) and (004)) and 70–90° ((110), (112), (006) and (201)) are typical for all carbon materials, and thus might indicate the presence of both 2D nanostructures, i.e., graphene oxide (GO) and reduced graphene oxide (rGO) [23,37], and other carbon materials. Similar XRD patterns were reported for various carbon materials, e.g., carbon aerogel [38], carbon black [39], carbon nanospheres [40] and activated carbon [41]. The crystallite size changed only slightly from 9.8 to 8.99 nm for anatase and from ca. 10.5 to 10.9 nm for rutile, suggesting that graphene did not only inhibit anatase-to-rutile transition, but also influenced the formation and growing of crystals, as already reported [22,42]. Interestingly, slight shift of anatase peaks to 25.26° for TiO_2_(C) and 25.27° for G-TiO_2_(C) samples ((101) anatase at 25.31°) might confirm C-doping for both samples (Figure 4c; in comparison to reference titania sample: P25 for clarity).

The Raman spectra for both samples were very similar, as shown in Figure 5, and a mapping recorded on a G-TiO_2_(C) sample is provided in Figure S1. Four clear peaks appeared at ca. 136, 390, 510 and 630 cm^−1^, corresponding well to E_g_, B_1g(1)_, A_1g_ + B_1g(2)_ and E_g(2)_ modes of anatase [19,20,21,43], respectively. Unfortunately, it was difficult to detect two characteristic peaks for graphene-kind structure at ca. 1304–1349 cm^−1^ and 1588–1601 cm^−1^ (as reported for graphene, GO, rGO, multiwall carbon nanotubes (MWCNT) of graphitic nature [19,20,23,44]), probably due to its low content. Interestingly, low-intensity peaks at ca. 1350 and 1560 cm^−1^ could be observed for TiO_2_(C) sample (Figure 5), which might result from surface modification of titania with carbon, as suggested by DRS spectrum (Figure 3a). Similar Raman spectra were reported for anatase titania NPs uniformly distributed inside the porous carbon matrix, synthesized using furfuryl alcohol, tetrabutyltitanate, and nonionic surfactant [45]. It is known that D and G bands of carbon structures are located at ca. 1350 and 1600 cm^−1^, and G band is common for all sp2 carbon forms giving information on the in-plane vibration of sp2 bonded carbon (tangential stretching mode of C=C bond), whereas D band is associated with structural disorder (the presence of sp3 defects) [19,44]. Therefore, it was confirmed that TiO_2_(C) was surface modified with carbon species, correlating well with high absorption at vis range (Figure 3a). Moreover, slight broadening of peaks (confirming a decrease in crystallite size (XRD data; Table 1)) and frequency shift after titania modification with graphene might be attributed to photon confinement, non-stoichiometry and internal stress/surface tension effects [45], which corresponds well with higher content of Ti^3+^ and electron traps (ETs) in G-TiO_2_(C) sample (as discussed further).

The surface of photocatalysts was characterized by XPS analysis, and obtained data are shown in Table 2 and Figure 6 Since both samples (TiO_2_(C) and G-TiO_2_(C)) were modified with carbon (during pyrolysis and annealing), pure titania sample (P25) was also analyzed for comparison and discussion. The ratio of oxygen to titanium exceeded stoichiometric value of 2.0, reaching 2.97 for P25, 5.7 for TiO_2_(C) and 3.38 for G-TiO_2_(C). Enrichment of titania surface with oxygen has been commonly reported as a result of adsorption of water/hydroxyl groups and carbon dioxide from air. For example, O/Ti ratios of 2.5 [46], 2.1–5.0 [47], 4.6 [48] and 7.7 [49] were reported for titania samples prepared by laser ablation, hydrothermal reaction, microemulsion and gas-phase methods, respectively. This is also confirmed by deconvolution of oxygen peak (Table 2) showing high content of hydroxyl groups on the titania surface. The binding energies of carbon, oxygen and titanium were estimated after deconvolution of C 1s, O 1s and Ti 2p_3/2_ peaks into three, three and two peaks, respectively, according to published reports on titania and carbon-modified titania samples [19,24,50,51,52]. Titanium was present mainly in Ti^4+^ form (TiO_2_(C)) and only low content of reduced titanium (Ti^3+^) was detected, i.e., 0.4%, 0.64% and 1.74% in P25, TiO_2_(C) and G-TiO_2_(C) samples, respectively. Almost all titania samples contain crystalline defects, observed mainly as Ti^3+^ form. It is proposed that graphene presence during titania synthesis disturbed in the formation of perfect titania crystals, and thus three times higher content of Ti^3+^ was noticed than that in TiO_2_(C) sample prepared in the absence of graphene sheets. In contrast to titanium spectra, the significant differences between samples were observed for carbon and oxygen peaks. In the case of titania, oxygen peak might be usually deconvoluted into three peaks, i.e., (i) oxygen in crystal lattice of TiO_2_ (at ca. 529.3 eV), (ii) C = O, Ti_2_O_3_ and OH groups bound to two titanium atoms (at ca. 531.7 eV) and (iii) hydroxyl groups bound to carbon or titanium (C–OH and Ti–OH; at 533.2 eV) [52]. In the case of graphene-modified samples, deconvolution of oxygen into two peaks was reported, i.e., (i) at 532.3 and 531.3 eV for lattice oxygen (Zn-O-Fe for Fe_2_O_3_-ZnO photocatalyst) and O–C bond in rGO, respectively [24], and (ii) at 529.7 and 531.4 eV for lattice oxygen (TiO_2_) and oxygen in adsorbed hydroxyl groups, respectively [19]. It is clear that oxygen in P25 was mainly in the form of TiO_2_ and water or/and carbon dioxide was also adsorbed on titania surface. However, in the case of carbon-modified samples, significant adsorption of oxygen (with different forms) on titania surfaces was noticed with different intensities, i.e., more organic carbon, C–OH and Ti–OH for TiO_2_(C) sample and C=O in G-TiO_2_(C) sample. Accordingly, it was confirmed that TiO_2_(C) sample was surface modified with carbon (from TTIP and/or C_2_H_4_), whereas G-TiO_2_(C) contained graphene-like forms of carbon (graphene could be partially oxidized during annealing). The presence of carbon is typical for all titania (and other oxides) samples, mainly due to carbon dioxide adsorption from surrounding air during sample preparation, which further forms bicarbonate and mono- and bidentate carbonate adsorbed on titania surface [53]. Of course, carbon from organic precursor of TiO_2_ (e.g., TTIP, titanium butoxide) might be also present in the final product (as also probable in TiO_2_(C) and G-TiO_2_(C) samples). It was found that samples prepared by pyrolysis contained much larger content of carbon on the surface than P25, i.e., ratio of C/Ti reached 3.6, 6.39 and 15.22 for P25, G-TiO_2_(C) and TiO_2_(C) samples, respectively, confirming significant enrichment of titania surface with carbon, especially for TiO_2_(C) sample (as suggested from DRS, XRD and Raman). In the case of titania samples, carbon might be deconvoluted also into three peaks indicating the presence of C–C, C–O and C=O at ca. 284.4, 286.1 and 288.6 eV, respectively. However, in the case of graphene-modified samples, deconvolution of carbon peak into two peaks was reported, i.e., (i) at 284.6 and 285.3 eV for graphitic carbon (C–C) and rGO (C–OH) [24], and (ii) at 284.5 and 288 eV for C–C/C–H bonds and C=O, respectively [19]. Here, carbon peak could be deconvoluted into three peaks for all samples, but the content of C–O and C=O was the highest in TiO_2_(C) and G-TiO_2_(C) samples, respectively. Therefore, it was concluded that adsorbed carbon (from TTIP and/or C_2_H_4_ or graphene) on titania surface was partly oxidized during annealing.

Since only surface characterization and crystalline composition could be obtained from XPS and XRD analyses, respectively, EDS measurement was additionally carried out for detailed samples’ characterization, and obtained data are shown in Figure 7. The large content of carbon was found in both samples, reaching 21.8 wt% and 51.0 wt% for TiO_2_(C) and G-TiO_2_(C) samples, respectively, confirming samples’ enrichment with carbon both on the surface (XPS data; Table 2) and in the bulk (crystalline (XRD data; Table 1) and amorphous carbon). Interestingly, it was found that the molar ratios of carbon to titanium differed significantly between samples, and between EDS and XPS results for TiO_2_(C) sample, i.e., C/Ti = 15.3 (XPS) and 1.6 (EDS) for TiO_2_(C) and Ti/C = 6.4 (XPS) and 6.3 (EDS) for G-TiO_2_(C). These differences suggest that graphene-modified sample is quite uniform in composition (bulk/surface), whereas carbon-modified sample (TiO_2_(C)) is mainly surface-modified with carbon. Therefore, it has been found that graphene co-presence during titania synthesis disturbs in titania surface modification with carbon.

For detail characterization of electronic properties of samples, RDB-PAS analysis was carried out for all samples, and obtained data are shown in Figure 8. It has been clarified that ERDT/CBB patterns might be used as a fingerprint of semiconducting metal-oxides powders for their identification [27,54]. This is because the CBB position, and ERDT pattern and total density of ETs (estimated from PAS and shown inside brackets in the Figure as “<ETs>”) might reflect bulk structure, surface structure and bulk/surface size, respectively. The trend of an increase in <ETs> along with an increase in specific surface area for commercial titania samples [52] suggests that measured ETs are predominantly located on the surface of particles with similar area density of ca. 1 ET per nm^2^. The peak of ERDT pattern of G-TiO_2_(C) was shifted to the low-energy, comparing to TiO_2_(C). Moreover, it appears that larger content of deep (ca. 2–3 eV) ETs was observed in TiO_2_(C) sample. On the other hand, the CBB positions were almost the same for both samples. Therefore, it is proposed that the modification with graphene could reduce the density of deep ETs (usually considered as recombination centers), but did not influence the band-gap energy. However, it must be pointed out that both samples could absorb visible light because the baseline of RDB-PA intensity in the vis range was much higher than that for commercial titania samples [27], as shown in Figure S2. Lower CBB position of P25 corresponds well with the presence of rutile of narrower bandgap than that in anatase, i.e., ca. 3.0 vs 3.2 eV, respectively. Therefore, it was concluded that P25 was not best sample for discussion of RDB-PAS data, since TiO_2_(C) and G-TiO_2_(C) contained mainly anatase phase. Accordingly, another titania sample (ST01; from Ishihara company) containing only anatase of fine NPs (ca. 10 nm) was analyzed. The CBB positions of G-TiO_2_(C) and TiO_2_(C) were nearly corresponding to that of ST01, confirming bandgap of anatase (CBB position seems to be almost constant since the bulk structure, i.e., anatase, is the same for TiO_2_(C), G-TiO_2_(C) and ST01). Interestingly, it was found that the total density of ETs (<ETs>)) was much lower in the samples prepared by laser synthesis than that in commercial titania samples (P25 and ST01) of similar properties (an increase in specific surface area results in an increase in the content of ETs, as discussed above [55]). Therefore, it was concluded that carbon from titania synthesis (TTIP and C_2_H_4_) might fill some crystalline defects, as proved by slight bandgap narrowing by DRS and XRD (Figure 3c,d and Figure 4c). Moreover, the observed higher-energy peaks at ca. 3.5 eV for TiO_2_(C) and G-TiO_2_(C) (inside CB), compared to those of P25 and ST01, indicate that the surfaces of TiO_2_(C) and G-TiO_2_(C) samples have amorphous nature, as has been observed for commercial amorphous titania and brayed samples (unpublished data), which might be induced by modification of sample with graphene.

### 3.2. Photocatalytic Activity

**Figure 9:**
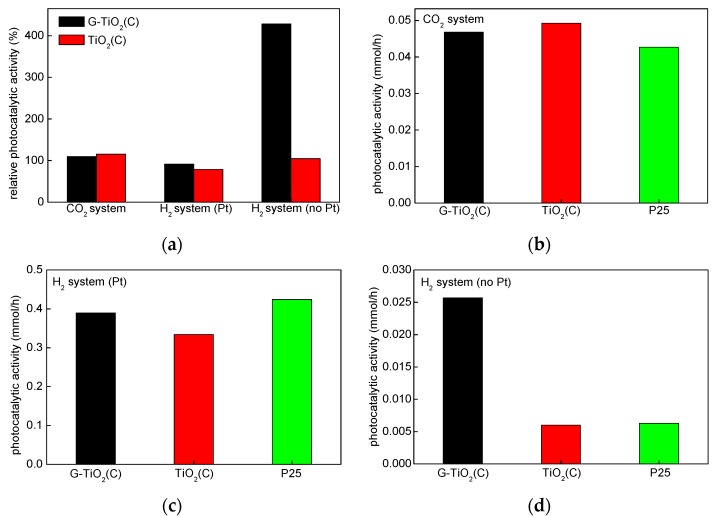
Photocatalytic activity data for: (**a**) relative activity in respect to that by P25 (100%) in three reaction systems; (**b**) oxidative decomposition of acetic acid (CO_2_ system); (**c**) dehydrogenation of methanol with in situ platinum deposition (H_2_ system (Pt)) and (**d**) dehydrogenation of methanol without in situ platinum deposition (H_2_ system (no Pt)).

**Figure 10:**
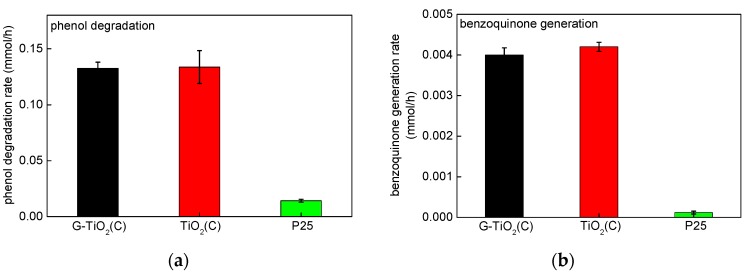
Photocatalytic activity under vis irradiation showing: (**a**) disappearance of phenol and (**b**) subsequent formation of benzoquinone during phenol degradation.

The photoactivity of samples was measured for oxidative decomposition of acetic acid under UV/vis (CO_2_ system), dehydrogenation of methanol under UV/vis (H_2_ system (Pt) and H_2_ system (no Pt)) and phenol degradation under vis. The obtained data are shown in Figure 9 and Figure 10. The comparison of activity under UV/vis for all systems to activity of reference sample: P25 (relative activity; activity of P25 = 100%) is shown in Figure 9a, whereas real activity data are shown in Figure 9b,c.

At first, data from Figure 9b,c were discussed, i.e., CO_2_ system and H_2_ system (Pt), since usually for anaerobic alcohol dehydrogenation, metallic co-catalyst (here Pt) must be used since titania is hardly active for hydrogen evolution. It was found that obtained photocatalysts exhibited high activity, similar to that by P25 in both reaction systems (oxidation and reduction), which is quite rare. Titania P25 (Degussa P25/Evonik P25/Aeroxide P25) is probably the most famous mixed-phase titania, due to extremely high photocatalytic activity in various photocatalytic reactions. For example, P25 was used for decomposition of organic and inorganic compounds present in water and wastewater [56,57], degradation of gas-phase pollutants [58], allergens’ removal [59], inactivation of microorganisms [60,61,62], self-cleaning surfaces [63,64] and solar energy conversion [65]. P25 is a white powder with fine NPs (ca. 30 nm), the density of ca. 3.9 g cm^−3^ and specific surface area of ca. 50 m^2^ g^−1^ [29,32]. The different composition of P25 might be found in the literature, i.e., 70–85% anatase and 15%–30% rutile, and the content of amorphous phase is usually not considered [66,67,68]. Our previous study indicated that P25 was not homogeneous samples, and the phase composition could vary between each P25 samples. Even P25 powders sampled from the same container possessed different content of anatase, rutile and amorphous phase, i.e., 73–85%, 13–17% and 0–13%, respectively [29,32], which was not surprising since the composition depended on the flame conditions (and even the location of formed particles in the flame) as P25 is produced by gas-phase flame synthesis. The comparison of P25 with 34 titania photocatalysts showed that P25 possessed one of the highest photocatalytic activity despite not the best properties (specific surface area, crystallite size, ETs content, crystallinity, etc.) [33]. It was reported that the photocatalytic efficiency did not depend only on the titania properties (specific surface area, polymorphic composition, defects’ content, crystallite and particle sizes), but also on the kind of photocatalytic reaction. Accordingly, it was proposed that: (i) large particle size resulted in efficient oxygen evolution, (ii) large specific surface area (small crystallite and particle sizes) in methanol dehydrogenation (H_2_ system), (iii) anatase presence in oxidative decomposition of acetic acid (CO_2_ system), (iv) rutile presence and defects’ content in oxidative decomposition of acetaldehyde and (v) rutile presence in organic synthesis. In our previous study, the higher activity in both reaction systems (CO_2_ and H_2_) than that by P25 was only obtained for faceted anatase sample of decahedral shape (decahedral anatase particles, DAPs [69]) with intrinsic properties of charge carriers’ separation, i.e., the migration of electrons to {101} and holes to {001} facets [70,71].

Accordingly, similar activity of P25 to that by TiO_2_(C) and G-TiO_2_(C) (and even slightly lower in CO_2_ system) indicates that both samples, prepared by laser synthesis, might be efficiently used for various photocatalytic reactions. Moreover, it should be pointed that even their color (greyish) does not significantly disturb in the overall activity (“inner-filter” effect). Slightly higher activity of TiO_2_(C) in CO_2_ system and G-TiO_2_(C) in H_2_ system (with Pt) suggests that the form of carbon governs photocatalytic action, and thus graphene is more recommended for hydrogen evolution. Indeed, graphene-like nanostructures have been known for high conductivity, participating in electron transfer [37], i.e., from CB of the semiconductor via G/GO/rGO to adsorbed molecules/compounds [20,21,22,23]. Interestingly, slightly higher activity of TiO_2_(C) than G-TiO_2_(C) and P25 for acetic acid oxidation should be discussed in consideration of photogenerated holes. Buchalska et al. reported that reactive oxygen species (ROS) generated by reaction of photogenerated holes with water or/and surface hydroxyl groups were mainly responsible for oxidation reaction (rather than ROS generated by reaction of photogenerated electrons with adsorbed oxygen) in the case of anatase samples [72]. Therefore, it is proposed that high enrichment of TiO_2_(C) surface with hydroxyl groups (either C–OH or Ti–OH (Figure 6 (center)) might cause an efficient formation of ROS, and thus high activity in oxidation reactions. Interestingly, it was found that in the case of methanol dehydrogenation in the absence of platinum, G-TiO_2_(C) sample was ca. five times more active than P25 and TiO_2_(C) sample (Figure 9d), suggesting that graphene-like structure might work as a co-catalyst for hydrogen generation.

Since photocatalysts were colored (greyish), the photocatalytic activity under vis irradiation was also tested for oxidative decomposition of phenol as a model compound. It should be considered that a disappearance of phenol (a decrease in its concentration in water) is not always equal with its degradation (possibility of adsorption on the photocatalyst surface), and thus the determination of oxidation products is recommended. Consequently, activity data were presented for both: (i) a decrease in the phenol concentration (Figure 10a) and (ii) formation of main intermediate, i.e., benzoquinone (Figure 10b). Indeed, it was found that both samples, prepared by pyrolysis, exhibited vis activity. The most active was TiO_2_(C), but an activity of G-TiO_2_(C) was only slightly lower, indicating that graphene might disturb in high activity of other carbon species adsorbed on titania surface or C-doped titania. However, an activity of bare P25 was meaningless, especially considering benzoquinone formation (these results confirm that estimation of compound disappearance (here phenol) is not recommended, due to its adsorption on photocatalyst surface). In contrast to the activities under UV/vis, G-TiO_2_(C) and TiO_2_(C) samples possessed the superior vis-activity than P25, due to the absorption of visible light (Figure 3). However, it should be pointed that absorption of vis light by modified titania samples does not guarantee vis activity. There are many colorous titania samples, practically inactive under vis irradiation. Therefore, carbon modification during titania synthesis and post-thermal annealing results in formation of vis-active materials. For clarification of the main reason of vis activity (surface sensitization, C-doping, etc.), an action spectrum analysis should be performed (planned further study).

**Figure 11:**
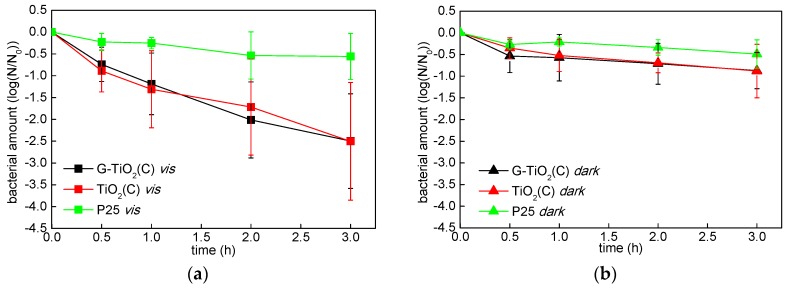
Bactericidal activities of G-TiO_2_(C) (black), TiO_2_(C) (red) and P25 (green) under: (**a**) vis irradiation and (**b**) dark conditions; data are expressed as mean ± SD (*n* = 3).

Since high photocatalytic activities of G-TiO_2_(C) and TiO_2_(C) under vis irradiation was shown, the antibacterial activity was examined under vis and in the dark condition (Figure 11). The antibacterial activities of G-TiO_2_(C) and TiO_2_(C) were almost same (considering experimental error) both under vis (Figure 11a) and in the dark (Figure 11b), i.e., relatively high activity under vis and low in the dark. Therefore, it is suggested that both samples showed the photocatalytic bactericidal activity, possibly due to the presence of carbon, rather than the existence of graphene. On the other hand, the activity of P25 was negligible both under vis and in the dark conditions, due to its wide band-gap and “dark” inertness. Some reports proposed that vis-responsive bactericidal activity of carbon-modified (mainly doped) titania [13,73,74,75,76,77] was cased by the presence of carbon and the direct interaction (e.g., redox reaction) between photocatalyst and bacteria. Interestingly, Wang et al. proposed that the electron transfer between C-doped titania nanotubes and bacteria induced the intracellular ROS formation and cell death, evaluated by charging of sample [78]. Moreover, Cruz-Ortiz et al. reported that TiO_2_–rGO showed significant bactericidal activity (*E. coli*) under visible light irradiation, due to the generation of singlet oxygen [79]. Accordingly, Markowska et al. found enhanced generation of hydroxyl radicals and decrease in cell viability (via activity of antioxidant enzymes, morphology change and mineralization) on glucose-modified titania under artificial solar irradiation [15]. Therefore, our results confirm high activity of carbon-modified samples under vis irradiation for bacteria inactivation, probably via generated ROS.

## 4. Conclusions

Laser pyrolysis with post-annealing proved to be an efficient method to obtain carbon-modified titania sample (TiO_2_(C)) with high photocatalytic activity under both UV and vis irradiation. Two sources of carbon might be considered, i.e., from titanium isopropoxide (TTIP; titania precursor) and C_2_H_4_ (a sensitizer used for laser pyrolysis). The modification of synthesis by addition of 0.04 wt% graphene into TTIP solution resulted in preparation of graphene/carbon-modified titania (G-TiO_2_(C)) with similar properties to non-modified sample (TiO_2_(C)). Although, all properties and activities of these two samples were similar, G-TiO_2_(C) showed ca. five times higher activity for methanol dehydrogenation without Pt co-catalyst under UV/vis irradiation than TiO_2_(C) and commercial titania sample (P25), indicating that graphene might be an efficient co-catalyst for hydrogen evolution. Similar activities of both samples for other reactions, i.e., oxidative decomposition of acetic acid under UV/vis, methanol dehydrogenation under UV/vis with Pt deposition in situ, phenol oxidation under vis and *E. coli* inactivation under vis, indicate that such low content of graphene (2.7 wt%) did not have significant influence on the overall activity. In contrast, carbon-modification seemed to be highly beneficial for activities in all systems since TiO_2_(C) and G-TiO_2_(C) samples exhibited similar activity to one of the most active commercial titania samples (P25) under UV/vis irradiation, and few orders of magnitude higher activity than P25 under vis irradiation. Therefore, simple modification with carbon allowed preparation of highly active and cheap (carbon modified) photocatalysts, active at broad range of irradiation, and thus being attractive alternative for other titania materials for environmental application.

## Figures and Tables

**Table 1:** Crystalline properties of samples.

Samples	Crystalline Content (%)				Crystallite Size (nm)			
	Anatase	Rutile	Carbon	Graphene	Anatase	Rutile	Carbon	Graphene
G-TiO_2_(C)	79.2	3.6	14.5	2.7	9.0	10.9	2.2	3.9
TiO_2_(C)	78.7	4.3	17.0	−	9.8	10.5	1.9	−

**Table 2:** Surface composition of samples determined by XPS analysis for C 1s, O 1s and Ti 2p3/2.

Samples	Content (at%)			C 1s (%)			O 1s (%)			Ti 2p_3/2_ (%)	
	C 1s	O 1s	Ti 2p_3/2_	C–C/C–H	C–O	C = O	TiO_2_	=O/–OH ^a^	–OH/C–O ^b^	Ti^3+^	Ti^4+^
G-TiO_2_(C)	59.30	31.42	9.28	55.85	11.35	32.80	43.70	37.11	19.20	1.74	98.26
TiO_2_(C)	69.44	26.00	4.56	48.45	38.97	12.58	25.53	23.93	50.54	0.64	99.36
P25	47.08	39.73	13.19	64.87	23.27	11.86	58.37	20.94	20.69	0.40	99.60

=O/–OH ^a^: Ti–(OH)–Ti, Ti_2_O_3_, C=O; –OH/C–O ^b^: Ti–OH, C–OH, organic carbon.

## Supplementary Materials

The following are available online at https://www.mdpi.com/1996-1944/12/24/4158/s1, Figure S1: Raman mapping of the G-TiO_2_(C) sample illustrating the presence of C species in localized places; Figure S2: RDB-PA spectra of (a) G-TiO_2_(C), (b) TiO_2_(C), (c) P25 (anatase/rutile), and (d) ST01 (anatase).
