# Supplementary material for: Carbon/Graphene-Modified Titania with Enhanced Photocatalytic Activity under UV and Vis Irradiation
Source: Materials (Basel). 2019 Dec 11;12(24):4158. doi: 10.3390/ma12244158 (PMC6947090; doi:10.3390/ma12244158)
Supplement: Supplementary file 1 [file materials-12-04158-s001.pdf]

materials

Electronic supplementary information for

Carbon/Graphene-Modified Titania with Enhanced Photocatalytic Activity under UV and Vis Irradiation

Kunlei Wang [1], Maya Endo-Kimura [1], Raphaëlle Belchi [2,3], Dong Zhang [4], Aurelie Habert [2], Johann Bouclé [3], Bunsho Ohtani [1,4], Ewa Kowalska [1,4,*] and Nathalie Herlin-Boime [2,*]

[1] Institute for Catalysis (ICAT), Hokkaido University, N21 W10, Sapporo 001-0021, Japan; kunlei@cat.hokudai.ac.jp (K.W.); m_endo@cat.hokudai.ac.jp (M.E.-K.); ohtani@cat.hokudai.ac.jp (B.O.)
[2] IRAMIS—NIMBE UMR 3685, Université Paris Saclay, CEA Saclay, 91191 Gif/Yvette CEDEX, France; raphaelle.belchi@cea.fr (R.B.); aurélie.habert@cea.fr (A.H.)
[3] Univ. Limoges, CNRS, XLIM, UMR 7252, F-87000 Limoges, France; johann.boucle@unilim.fr
[4] Graduate School of Environmental Science, Hokkaido University, Sapporo 060-0810, Japan; zhang.d@cat.hokudai.ac.jp
* Correspondence: kowalska@cat.hokudai.ac.jp (E.K.); nathalie.herlin@cea.fr (N.H.-B.)

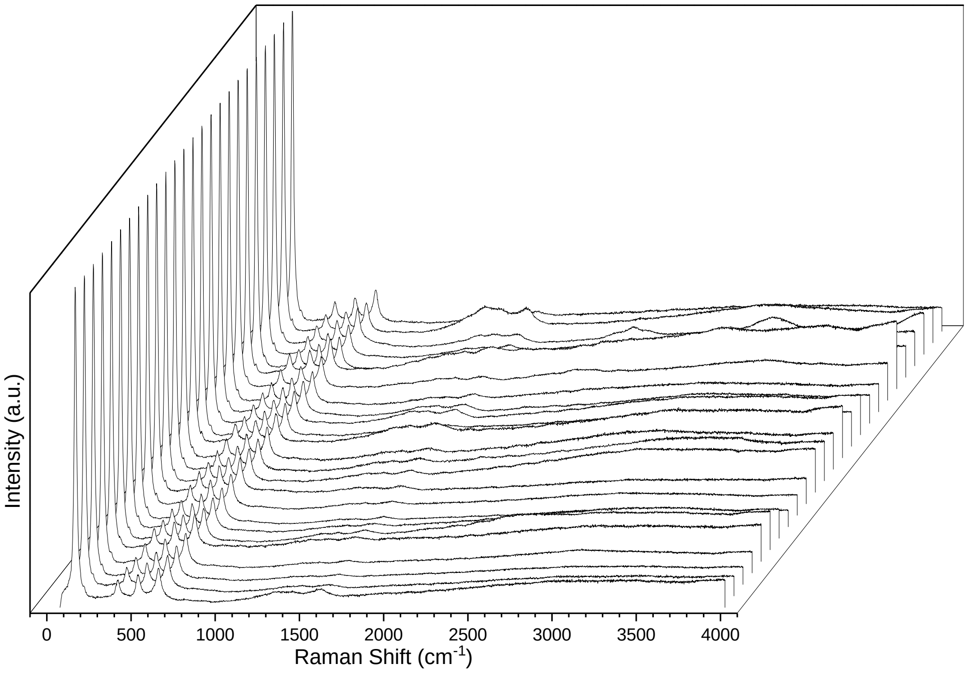

Figure S1. Raman mapping of the G-TiO_2 sample illustrating the presence of C species in localized places.

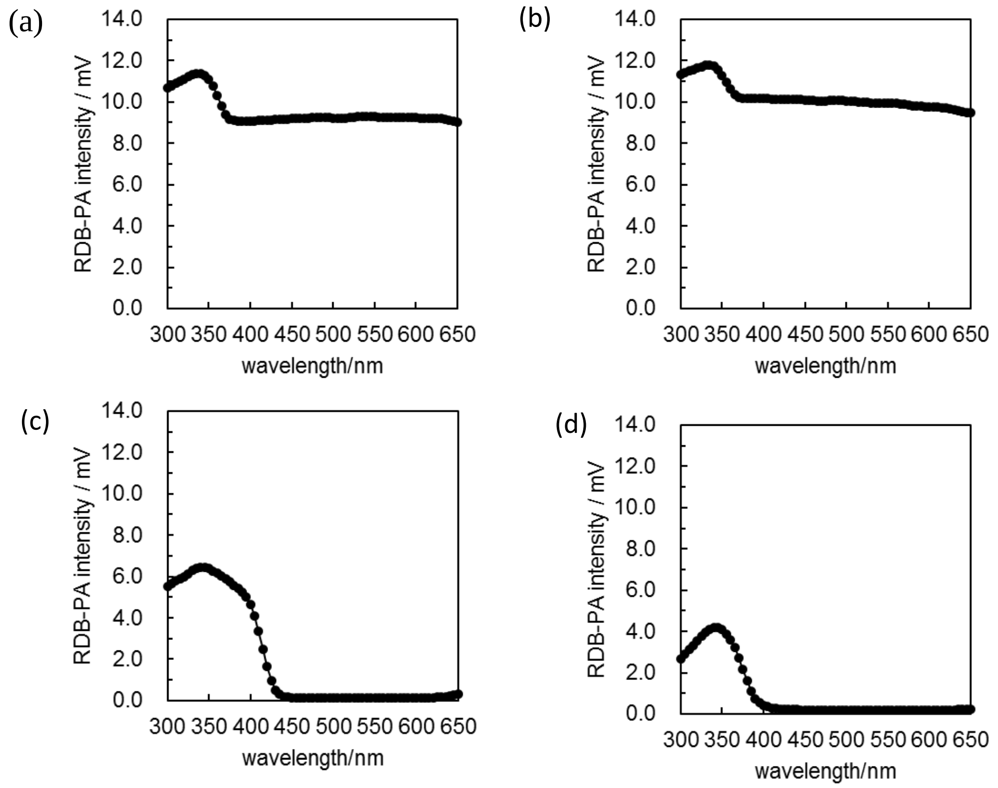

Figure S2. RDB-PA spectra of (a) G-TiO_2, (b) TiO_2, (c) P25 (anatase/rutile), and (d) ST01 (anatase).
